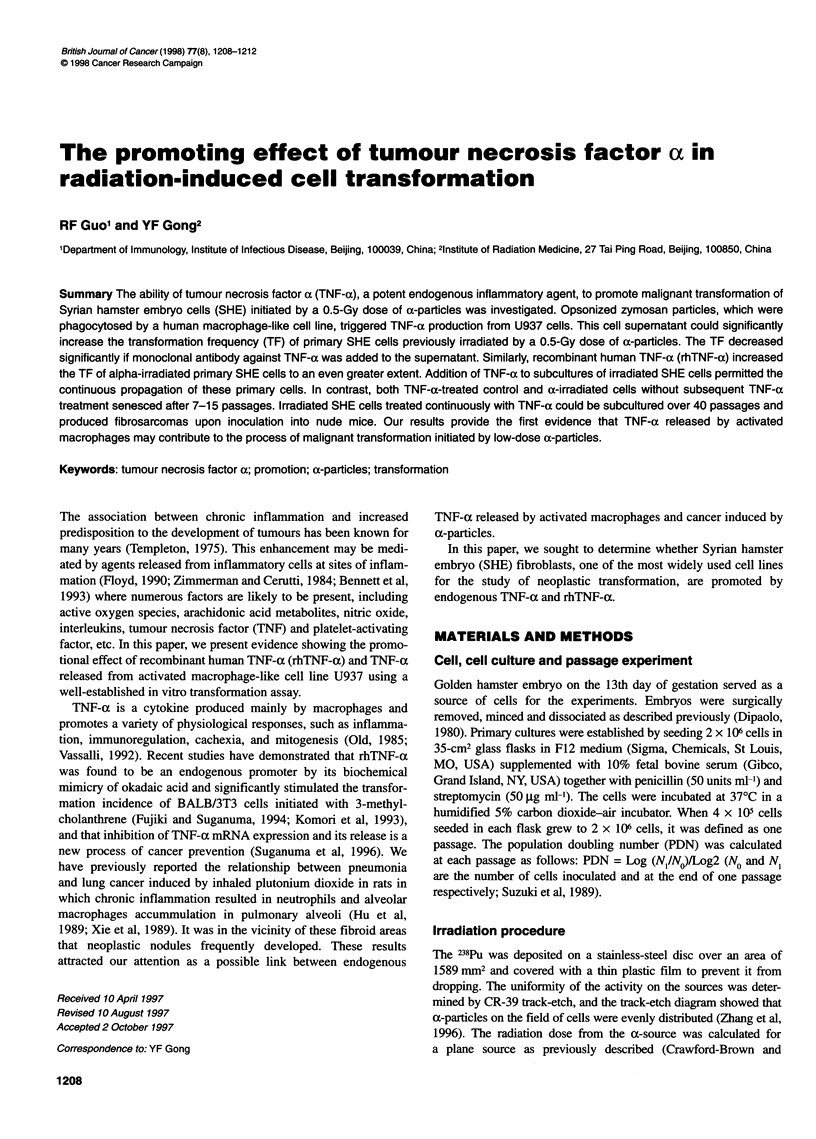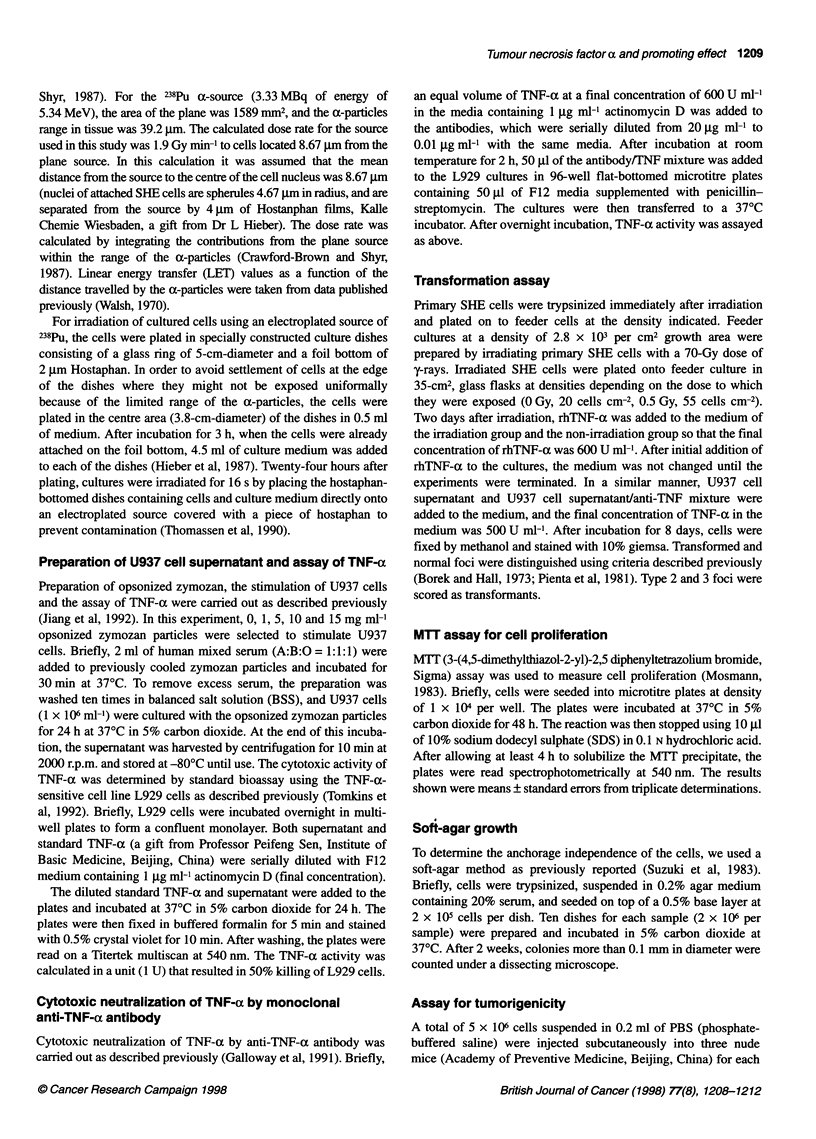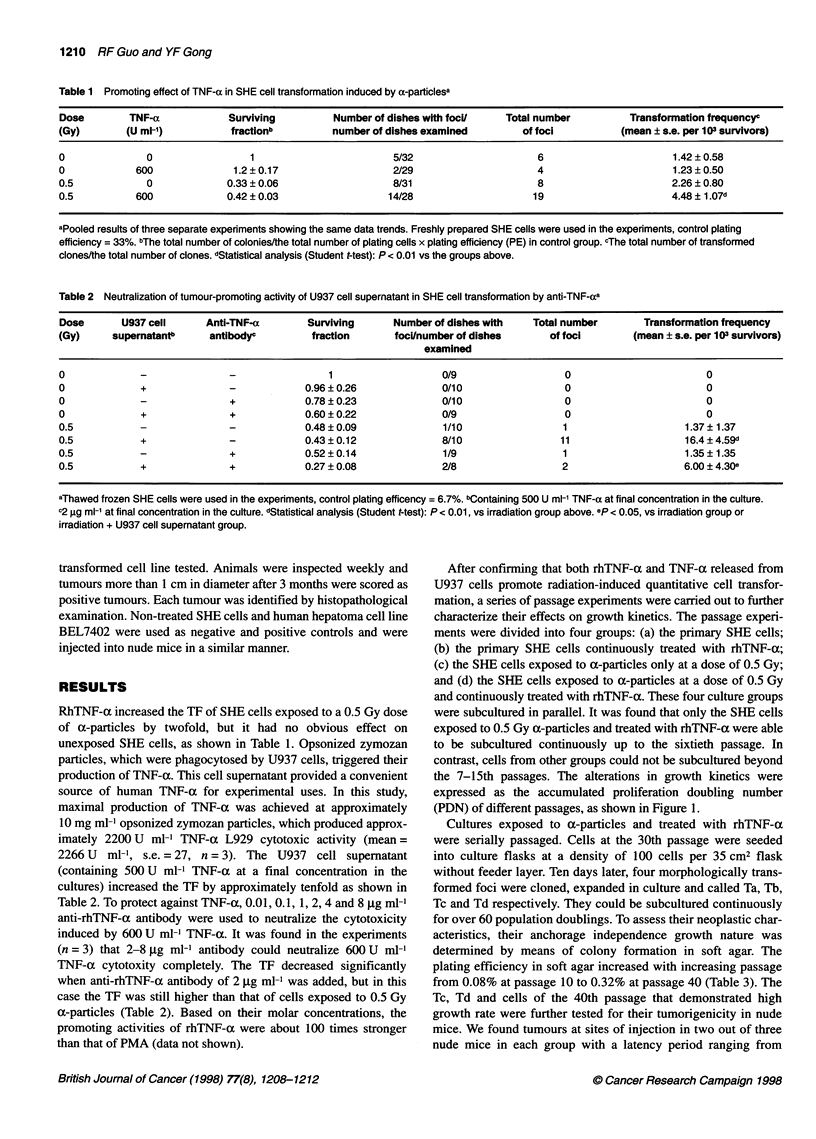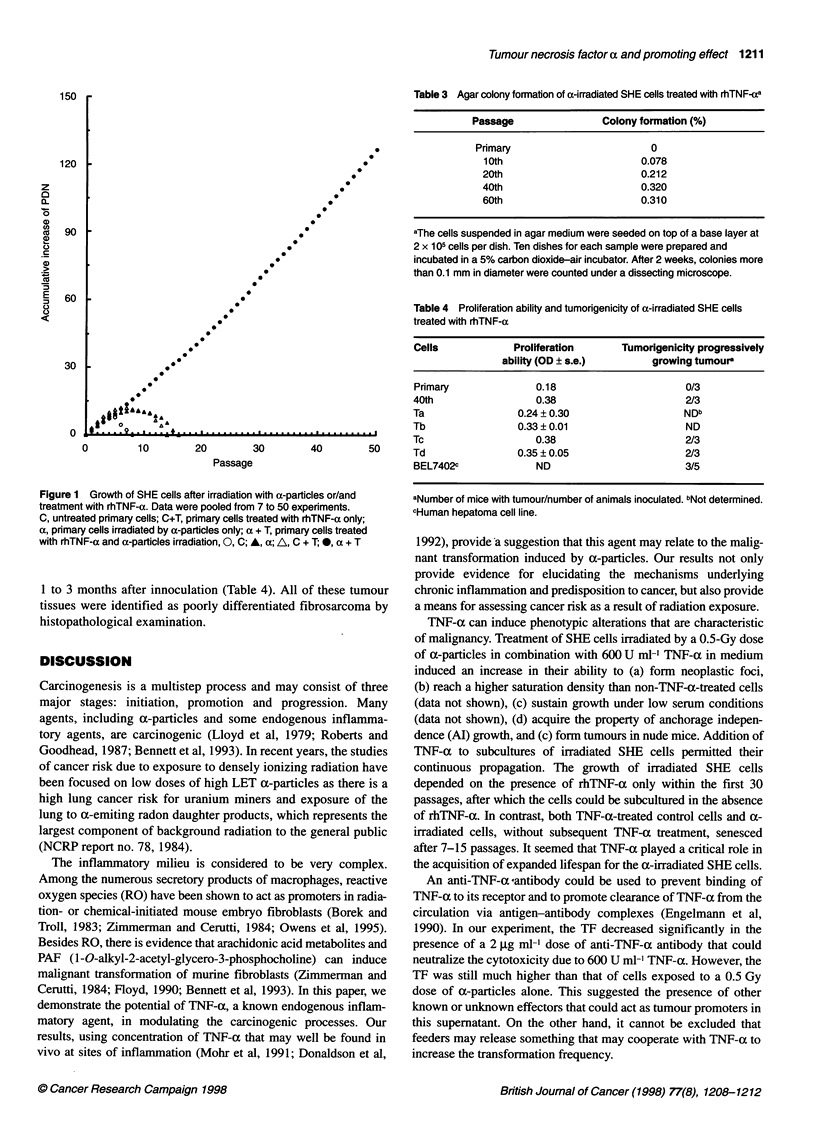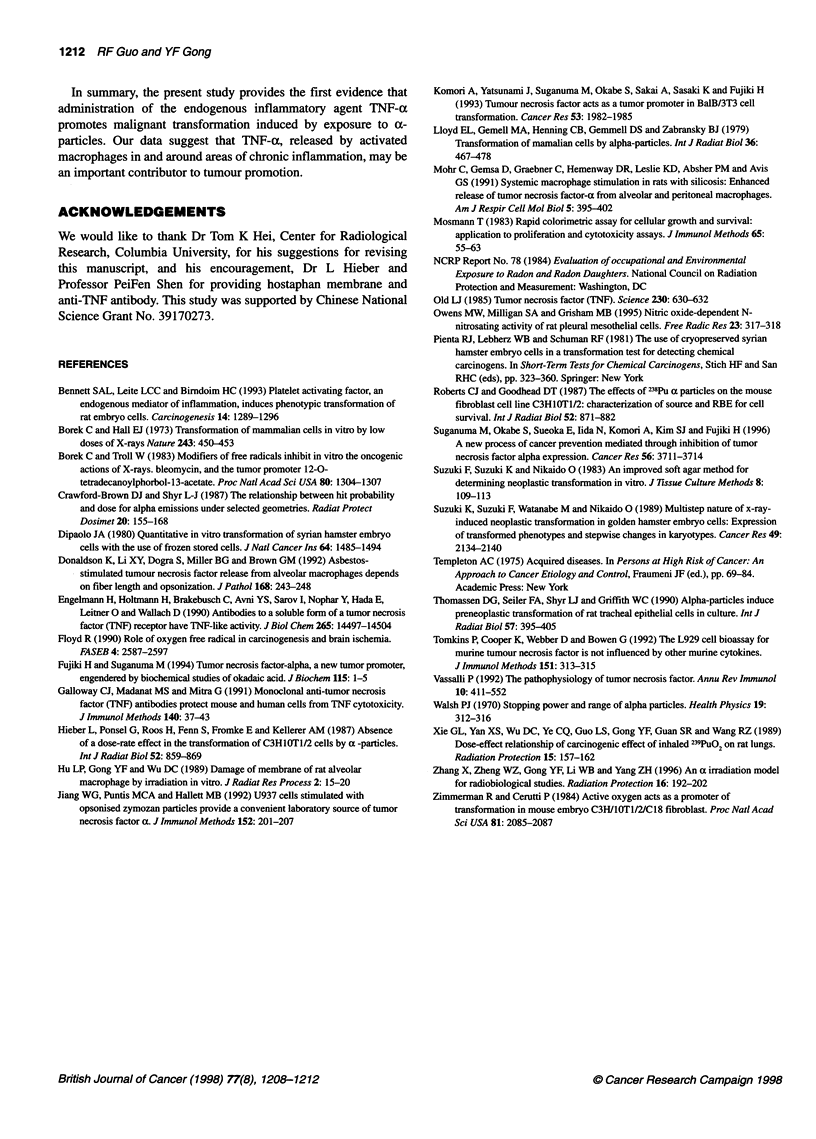# The promoting effect of tumour necrosis factor alpha in radiation-induced cell transformation.

**DOI:** 10.1038/bjc.1998.204

**Published:** 1998-04

**Authors:** R. F. Guo, Y. F. Gong

**Affiliations:** Department of Immunology, Institute of Infectious Disease, Beijing, China.

## Abstract

The ability of tumour necrosis factor alpha (TNF-alpha), a potent endogenous inflammatory agent, to promote malignant transformation of Syrian hamster embryo cells (SHE) initiated by a 0.5-Gy dose of alpha-particles was investigated. Opsonized zymosan particles, which were phagocytosed by a human macrophage-like cell line, triggered TNF-alpha production from U937 cells. This cell supernatant could significantly increase the transformation frequency (TF) of primary SHE cells previously irradiated by a 0.5-Gy dose of alpha-particles. The TF decreased significantly if monoclonal antibody against TNF-alpha was added to the supernatant. Similarly, recombinant human TNF-alpha (rhTNF-alpha) increased the TF of alpha-irradiated primary SHE cells to an even greater extent. Addition of TNF-alpha to subcultures of irradiated SHE cells permitted the continuous propagation of these primary cells. In contrast, both TNF-alpha-treated control and alpha-irradiated cells without subsequent TNF-alpha treatment senesced after 7-15 passages. Irradiated SHE cells treated continuously with TNF-alpha could be subcultured over 40 passages and produced fibrosarcomas upon inoculation into nude mice. Our results provide the first evidence that TNF-alpha released by activated macrophages may contribute to the process of malignant transformation initiated by low-dose alpha-particles.


British Journal of Cancer (1998) 77(8), 1208–1212
© 1998 Cancer Research Campaign

### The promoting effect of tumour necrosis factor α in radiation-induced cell transformation

**RF Guo[1] and YF Gong[2]**

[1]Department of Immunology, Institute of Infectious Disease, Beijing, 100039, China; [2]Institute of Radiation Medicine, 27 Tai Ping Road, Beijing, 100850, China

**Summary** The ability of tumour necrosis factor α (TNF-α), a potent endogenous inflammatory agent, to promote malignant transformation of Syrian hamster embryo cells (SHE) initiated by a 0.5-Gy dose of α-particles was investigated. Opsonized zymosan particles, which were phagocytosed by a human macrophage-like cell line, triggered TNF-α production from U937 cells. This cell supernatant could significantly increase the transformation frequency (TF) of primary SHE cells previously irradiated by a 0.5-Gy dose of α-particles. The TF decreased significantly if monoclonal antibody against TNF-α was added to the supernatant. Similarly, recombinant human TNF-α (rhTNF-α) increased the TF of alpha-irradiated primary SHE cells to an even greater extent. Addition of TNF-α to subcultures of irradiated SHE cells permitted the continuous propagation of these primary cells. In contrast, both TNF-α-treated control and α-irradiated cells without subsequent TNF-α treatment senesced after 7–15 passages. Irradiated SHE cells treated continuously with TNF-α could be subcultured over 40 passages and produced fibrosarcomas upon inoculation into nude mice. Our results provide the first evidence that TNF-α released by activated macrophages may contribute to the process of malignant transformation initiated by low-dose α-particles.

**Keywords**: tumour necrosis factor α; promotion; α-particles; transformation

The association between chronic inflammation and increased predisposition to the development of tumours has been known for many years (Templeton, 1975). This enhancement may be mediated by agents released from inflammatory cells at sites of inflammation (Floyd, 1990; Zimmerman and Cerutti, 1984; Bennett et al, 1993) where numerous factors are likely to be present, including active oxygen species, arachidonic acid metabolites, nitric oxide, interleukins, tumour necrosis factor (TNF) and platelet-activating factor, etc. In this paper, we present evidence showing the promotional effect of recombinant human TNF-α (rhTNF-α) and TNF-α released from activated macrophage-like cell line U937 using a well-established in vitro transformation assay.

TNF-α is a cytokine produced mainly by macrophages and promotes a variety of physiological responses, such as inflammation, immunoregulation, cachexia, and mitogenesis (Old, 1985; Vassalli, 1992). Recent studies have demonstrated that rhTNF-α was found to be an endogenous promoter by its biochemical mimicry of okadaic acid and significantly stimulated the transformation incidence of BALB/3T3 cells initiated with 3-methylcholanthrene (Fujiki and Suganuma, 1994; Komori et al, 1993), and that inhibition of TNF-α mRNA expression and its release is a new process of cancer prevention (Suganuma et al, 1996). We have previously reported the relationship between pneumonia and lung cancer induced by inhaled plutonium dioxide in rats in which chronic inflammation resulted in neutrophils and alveolar macrophages accummulation in pulmonary alveoli (Hu et al, 1989; Xie et al, 1989). It was in the vicinity of these fibroid areas that neoplastic nodules frequently developed. These results attracted our attention as a possible link between endogenous TNF-α released by activated macrophages and cancer induced by α-particles.

In this paper, we sought to determine whether Syrian hamster embryo (SHE) fibroblasts, one of the most widely used cell lines for the study of neoplastic transformation, are promoted by endogenous TNF-α and rhTNF-α.

*Received 10 April 1997*
*Revised 10 August 1997*
*Accepted 2 October 1997*

*Correspondence to:* YF Gong

### MATERIALS AND METHODS

### Cell, cell culture and passage experiment

Golden hamster embryo on the 13th day of gestation served as a source of cells for the experiments. Embryos were surgically removed, minced and dissociated as described previously (Dipaolo, 1980). Primary cultures were established by seeding $2 \times 10^6$ cells in 35-cm$^2$ glass flasks in F12 medium (Sigma, Chemicals, St Louis, MO, USA) supplemented with 10% fetal bovine serum (Gibco, Grand Island, NY, USA) together with penicillin (50 units ml$^{-1}$) and streptomycin (50 μg ml$^{-1}$). The cells were incubated at 37°C in a humidified 5% carbon dioxide–air incubator. When $4 \times 10^5$ cells seeded in each flask grew to $2 \times 10^6$ cells, it was defined as one passage. The population doubling number (PDN) was calculated at each passage as follows: PDN = Log $(N_1/N_0)$/Log2 ($N_0$ and $N_1$ are the number of cells inoculated and at the end of one passage respectively; Suzuki et al, 1989).

### Irradiation procedure

The $^{238}$Pu was deposited on a stainless-steel disc over an area of 1589 mm$^2$ and covered with a thin plastic film to prevent it from dropping. The uniformity of the activity on the sources was determined by CR-39 track-etch, and the track-etch diagram showed that α-particles on the field of cells were evenly distributed (Zhang et al, 1996). The radiation dose from the α-source was calculated for a plane source as previously described (Crawford-Brown and

Shyr, 1987). For the $^{238}Pu$ α-source (3.33 MBq of energy of 5.34 MeV), the area of the plane was 1589 mm$^2$, and the α-particles range in tissue was 39.2 µm. The calculated dose rate for the source used in this study was 1.9 Gy min$^{-1}$ to cells located 8.67 µm from the plane source. In this calculation it was assumed that the mean distance from the source to the centre of the cell nucleus was 8.67 µm (nuclei of attached SHE cells are spherules 4.67 µm in radius, and are separated from the source by 4 µm of Hostanphan films, Kalle Chemie Wiesbaden, a gift from Dr L Hieber). The dose rate was calculated by integrating the contributions from the plane source within the range of the α-particles (Crawford-Brown and Shyr, 1987). Linear energy transfer (LET) values as a function of the distance travelled by the α-particles were taken from data published previously (Walsh, 1970).

For irradiation of cultured cells using an electroplated source of $^{238}Pu$, the cells were plated in specially constructed culture dishes consisting of a glass ring of 5-cm-diameter and a foil bottom of 2 µm Hostaphan. In order to avoid settlement of cells at the edge of the dishes where they might not be exposed uniformally because of the limited range of the α-particles, the cells were plated in the centre area (3.8-cm-diameter) of the dishes in 0.5 ml of medium. After incubation for 3 h, when the cells were already attached on the foil bottom, 4.5 ml of culture medium was added to each of the dishes (Hieber et al, 1987). Twenty-four hours after plating, cultures were irradiated for 16 s by placing the hostaphan-bottomed dishes containing cells and culture medium directly onto an electroplated source covered with a piece of hostaphan to prevent contamination (Thomassen et al, 1990).

### Preparation of U937 cell supernatant and assay of TNF-α

Preparation of opsonized zymozan, the stimulation of U937 cells and the assay of TNF-α were carried out as described previously (Jiang et al, 1992). In this experiment, 0, 1, 5, 10 and 15 mg ml$^{-1}$ opsonized zymozan particles were selected to stimulate U937 cells. Briefly, 2 ml of human mixed serum (A:B:O = 1:1:1) were added to previously cooled zymozan particles and incubated for 30 min at 37°C. To remove excess serum, the preparation was washed ten times in balanced salt solution (BSS), and U937 cells (1 × 10$^6$ ml$^{-1}$) were cultured with the opsonized zymozan particles for 24 h at 37°C in 5% carbon dioxide. At the end of this incubation, the supernatant was harvested by centrifugation for 10 min at 2000 r.p.m. and stored at –80°C until use. The cytotoxic activity of TNF-α was determined by standard bioassay using the TNF-α-sensitive cell line L929 cells as described previously (Tomkins et al, 1992). Briefly, L929 cells were incubated overnight in multiwell plates to form a confluent monolayer. Both supernatant and standard TNF-α (a gift from Professor Peifeng Sen, Institute of Basic Medicine, Beijing, China) were serially diluted with F12 medium containing 1 µg ml$^{-1}$ actinomycin D (final concentration).

The diluted standard TNF-α and supernatant were added to the plates and incubated at 37°C in 5% carbon dioxide for 24 h. The plates were then fixed in buffered formalin for 5 min and stained with 0.5% crystal violet for 10 min. After washing, the plates were read on a Titertek multiscan at 540 nm. The TNF-α activity was calculated in a unit (1 U) that resulted in 50% killing of L929 cells.

### Cytotoxic neutralization of TNF-α by monoclonal anti-TNF-α antibody

Cytotoxic neutralization of TNF-α by anti-TNF-α antibody was carried out as described previously (Galloway et al, 1991). Briefly, an equal volume of TNF-α at a final concentration of 600 U ml$^{-1}$ in the media containing 1 µg ml$^{-1}$ actinomycin D was added to the antibodies, which were serially diluted from 20 µg ml$^{-1}$ to 0.01 µg ml$^{-1}$ with the same media. After incubation at room temperature for 2 h, 50 µl of the antibody/TNF mixture was added to the L929 cultures in 96-well flat-bottomed microtitre plates containing 50 µl of F12 media supplemented with penicillin–streptomycin. The cultures were then transferred to a 37°C incubator. After overnight incubation, TNF-α activity was assayed as above.

### Transformation assay

Primary SHE cells were trypsinized immediately after irradiation and plated on to feeder cells at the density indicated. Feeder cultures at a density of 2.8 × 10$^3$ per cm$^2$ growth area were prepared by irradiating primary SHE cells with a 70-Gy dose of γ-rays. Irradiated SHE cells were plated onto feeder culture in 35-cm$^2$, glass flasks at densities depending on the dose to which they were exposed (0 Gy, 20 cells cm$^{-2}$, 0.5 Gy, 55 cells cm$^{-2}$). Two days after irradiation, rhTNF-α was added to the medium of the irradiation group and the non-irradiation group so that the final concentration of rhTNF-α was 600 U ml$^{-1}$. After initial addition of rhTNF-α to the cultures, the medium was not changed until the experiments were terminated. In a similar manner, U937 cell supernatant and U937 cell supernatant/anti-TNF mixture were added to the medium, and the final concentration of TNF-α in the medium was 500 U ml$^{-1}$. After incubation for 8 days, cells were fixed by methanol and stained with 10% giemsa. Transformed and normal foci were distinguished using criteria described previously (Borek and Hall, 1973; Pienta et al, 1981). Type 2 and 3 foci were scored as transformants.

### MTT assay for cell proliferation

MTT (3-(4,5-dimethylthiazol-2-yl)-2,5 diphenyltetrazolium bromide, Sigma) assay was used to measure cell proliferation (Mosmann, 1983). Briefly, cells were seeded into microtitre plates at density of 1 × 10$^4$ per well. The plates were incubated at 37°C in 5% carbon dioxide for 48 h. The reaction was then stopped using 10 µl of 10% sodium dodecyl sulphate (SDS) in 0.1 N hydrochloric acid. After allowing at least 4 h to solubilize the MTT precipitate, the plates were read spectrophotometrically at 540 nm. The results shown were means ± standard errors from triplicate determinations.

### Soft-agar growth

To determine the anchorage independence of the cells, we used a soft-agar method as previously reported (Suzuki et al, 1983). Briefly, cells were trypsinized, suspended in 0.2% agar medium containing 20% serum, and seeded on top of a 0.5% base layer at 2 × 10$^5$ cells per dish. Ten dishes for each sample (2 × 10$^6$ per sample) were prepared and incubated in 5% carbon dioxide at 37°C. After 2 weeks, colonies more than 0.1 mm in diameter were counted under a dissecting microscope.

### Assay for tumorigenicity

A total of 5 × 10$^6$ cells suspended in 0.2 ml of PBS (phosphate-buffered saline) were injected subcutaneously into three nude mice (Academy of Preventive Medicine, Beijing, China) for each

© Cancer Research Campaign 1998

**Table 1** Promoting effect of TNF-α in SHE cell transformation induced by α-particles[a]

| Dose (Gy) | TNF-α (U ml$^{-1}$) | Surviving fraction[b] | Number of dishes with foci/ number of dishes examined | Total number of foci | Transformation frequency[c] (mean ± s.e. per $10^3$ survivors) |
|---|---|---|---|---|---|
| 0 | 0 | 1 | 5/32 | 6 | 1.42 ± 0.58 |
| 0 | 600 | 1.2 ± 0.17 | 2/29 | 4 | 1.23 ± 0.50 |
| 0.5 | 0 | 0.33 ± 0.06 | 8/31 | 8 | 2.26 ± 0.80 |
| 0.5 | 600 | 0.42 ± 0.03 | 14/28 | 19 | 4.48 ± 1.07[d] |

[a]Pooled results of three separate experiments showing the same data trends. Freshly prepared SHE cells were used in the experiments, control plating efficiency = 33%. [b]The total number of colonies/the total number of plating cells × plating efficiency (PE) in control group. [c]The total number of transformed clones/the total number of clones. [d]Statistical analysis (Student *t*-test): $P < 0.01$ vs the groups above.

**Table 2** Neutralization of tumour-promoting activity of U937 cell supernatant in SHE cell transformation by anti-TNF-α[a]

| Dose (Gy) | U937 cell supernatant[b] | Anti-TNF-α antibody[c] | Surviving fraction | Number of dishes with foci/number of dishes examined | Total number of foci | Transformation frequency (mean ± s.e. per $10^3$ survivors) |
|---|---|---|---|---|---|---|
| 0 | – | – | 1 | 0/9 | 0 | 0 |
| 0 | + | – | 0.96 ± 0.26 | 0/10 | 0 | 0 |
| 0 | – | + | 0.78 ± 0.23 | 0/10 | 0 | 0 |
| 0 | + | + | 0.60 ± 0.22 | 0/9 | 0 | 0 |
| 0.5 | – | – | 0.48 ± 0.09 | 1/10 | 1 | 1.37 ± 1.37 |
| 0.5 | + | – | 0.43 ± 0.12 | 8/10 | 11 | 16.4 ± 4.59[d] |
| 0.5 | – | + | 0.52 ± 0.14 | 1/9 | 1 | 1.35 ± 1.35 |
| 0.5 | + | + | 0.27 ± 0.08 | 2/8 | 2 | 6.00 ± 4.30[e] |

[a]Thawed frozen SHE cells were used in the experiments, control plating efficency = 6.7%. [b]Containing 500 U ml$^{-1}$ TNF-α at final concentration in the culture. [c]2 μg ml$^{-1}$ at final concentration in the culture. [d]Statistical analysis (Student *t*-test): $P < 0.01$, vs irradiation group above. [e]$P < 0.05$, vs irradiation group or irradiation + U937 cell supernatant group.

transformed cell line tested. Animals were inspected weekly and tumours more than 1 cm in diameter after 3 months were scored as positive tumours. Each tumour was identified by histopathological examination. Non-treated SHE cells and human hepatoma cell line BEL7402 were used as negative and positive controls and were injected into nude mice in a similar manner.

### RESULTS

RhTNF-α increased the TF of SHE cells exposed to a 0.5 Gy dose of α-particles by twofold, but it had no obvious effect on unexposed SHE cells, as shown in Table 1. Opsonized zymozan particles, which were phagocytosed by U937 cells, triggered their production of TNF-α. This cell supernatant provided a convenient source of human TNF-α for experimental uses. In this study, maximal production of TNF-α was achieved at approximately 10 mg ml$^{-1}$ opsonized zymozan particles, which produced approximately 2200 U ml$^{-1}$ TNF-α L929 cytotoxic activity (mean = 2266 U ml$^{-1}$, s.e. = 27, $n = 3$). The U937 cell supernatant (containing 500 U ml$^{-1}$ TNF-α at a final concentration in the cultures) increased the TF by approximately tenfold as shown in Table 2. To protect against TNF-α, 0.01, 0.1, 1, 2, 4 and 8 μg ml$^{-1}$ anti-rhTNF-α antibody were used to neutralize the cytotoxicity induced by 600 U ml$^{-1}$ TNF-α. It was found in the experiments ($n = 3$) that 2–8 μg ml$^{-1}$ antibody could neutralize 600 U ml$^{-1}$ TNF-α cytotoxity completely. The TF decreased significantly when anti-rhTNF-α antibody of 2 μg ml$^{-1}$ was added, but in this case the TF was still higher than that of cells exposed to 0.5 Gy α-particles (Table 2). Based on their molar concentrations, the promoting activities of rhTNF-α were about 100 times stronger than that of PMA (data not shown).

After confirming that both rhTNF-α and TNF-α released from U937 cells promote radiation-induced quantitative cell transformation, a series of passage experiments were carried out to further characterize their effects on growth kinetics. The passage experiments were divided into four groups: (a) the primary SHE cells; (b) the primary SHE cells continuously treated with rhTNF-α; (c) the SHE cells exposed to α-particles only at a dose of 0.5 Gy; and (d) the SHE cells exposed to α-particles at a dose of 0.5 Gy and continuously treated with rhTNF-α. These four culture groups were subcultured in parallel. It was found that only the SHE cells exposed to 0.5 Gy α-particles and treated with rhTNF-α were able to be subcultured continuously up to the sixtieth passage. In contrast, cells from other groups could not be subcultured beyond the 7–15th passages. The alterations in growth kinetics were expressed as the accumulated proliferation doubling number (PDN) of different passages, as shown in Figure 1.

Cultures exposed to α-particles and treated with rhTNF-α were serially passaged. Cells at the 30th passage were seeded into culture flasks at a density of 100 cells per 35 cm$^2$ flask without feeder layer. Ten days later, four morphologically transformed foci were cloned, expanded in culture and called Ta, Tb, Tc and Td respectively. They could be subcultured continuously for over 60 population doublings. To assess their neoplastic characteristics, their anchorage independence growth nature was determined by means of colony formation in soft agar. The plating efficiency in soft agar increased with increasing passage from 0.08% at passage 10 to 0.32% at passage 40 (Table 3). The Tc, Td and cells of the 40th passage that demonstrated high growth rate were further tested for their tumorigenicity in nude mice. We found tumours at sites of injection in two out of three nude mice in each group with a latency period ranging from

*© Cancer Research Campaign 1998*

**Figure 1** Growth of SHE cells after irradiation with α-particles or/and treatment with rhTNF-α. Data were pooled from 7 to 50 experiments. C, untreated primary cells; C+T, primary cells treated with rhTNF-α only; α, primary cells irradiated by α-particles only; α + T, primary cells treated with rhTNF-α and α-particles irradiation, ○, C; ▲, α; △, C + T; ●, α + T

1 to 3 months after innoculation (Table 4). All of these tumour tissues were identified as poorly differentiated fibrosarcoma by histopathological examination.

### DISCUSSION

Carcinogenesis is a multistep process and may consist of three major stages: initiation, promotion and progression. Many agents, including α-particles and some endogenous inflammatory agents, are carcinogenic (Lloyd et al, 1979; Roberts and Goodhead, 1987; Bennett et al, 1993). In recent years, the studies of cancer risk due to exposure to densely ionizing radiation have been focused on low doses of high LET α-particles as there is a high lung cancer risk for uranium miners and exposure of the lung to α-emiting radon daughter products, which represents the largest component of background radiation to the general public (NCRP report no. 78, 1984).

The inflammatory milieu is considered to be very complex. Among the numerous secretory products of macrophages, reactive oxygen species (RO) have been shown to act as promoters in radiation- or chemical-initiated mouse embryo fibroblasts (Borek and Troll, 1983; Zimmerman and Cerutti, 1984; Owens et al, 1995). Besides RO, there is evidence that arachidonic acid metabolites and PAF (1-*O*-alkyl-2-acetyl-glycero-3-phosphocholine) can induce malignant transformation of murine fibroblasts (Zimmerman and Cerutti, 1984; Floyd, 1990; Bennett et al, 1993). In this paper, we demonstrate the potential of TNF-α, a known endogenous inflammatory agent, in modulating the carcinogenic processes. Our results, using concentration of TNF-α that may well be found in vivo at sites of inflammation (Mohr et al, 1991; Donaldson et al, 1992), provide a suggestion that this agent may relate to the malignant transformation induced by α-particles. Our results not only provide evidence for elucidating the mechanisms underlying chronic inflammation and predisposition to cancer, but also provide a means for assessing cancer risk as a result of radiation exposure.

**Table 3** Agar colony formation of α-irradiated SHE cells treated with rhTNF-α[a]

| Passage | Colony formation (%) |
|---|---|
| Primary | 0 |
| 10th | 0.078 |
| 20th | 0.212 |
| 40th | 0.320 |
| 60th | 0.310 |

[a]The cells suspended in agar medium were seeded on top of a base layer at $2 \times 10^5$ cells per dish. Ten dishes for each sample were prepared and incubated in a 5% carbon dioxide–air incubator. After 2 weeks, colonies more than 0.1 mm in diameter were counted under a dissecting microscope.

**Table 4** Proliferation ability and tumorigenicity of α-irradiated SHE cells treated with rhTNF-α

| Cells | Proliferation ability (OD ± s.e.) | Tumorigenicity progressively growing tumour[a] |
|---|---|---|
| Primary | 0.18 | 0/3 |
| 40th | 0.38 | 2/3 |
| Ta | 0.24 ± 0.30 | ND[b] |
| Tb | 0.33 ± 0.01 | ND |
| Tc | 0.38 | 2/3 |
| Td | 0.35 ± 0.05 | 2/3 |
| BEL7402[c] | ND | 3/5 |

[a]Number of mice with tumour/number of animals inoculated. [b]Not determined. [c]Human hepatoma cell line.

TNF-α can induce phenotypic alterations that are characteristic of malignancy. Treatment of SHE cells irradiated by a 0.5-Gy dose of α-particles in combination with 600 U $ml^{-1}$ TNF-α in medium induced an increase in their ability to (a) form neoplastic foci, (b) reach a higher saturation density than non-TNF-α-treated cells (data not shown), (c) sustain growth under low serum conditions (data not shown), (d) acquire the property of anchorage independence (AI) growth, and (c) form tumours in nude mice. Addition of TNF-α to subcultures of irradiated SHE cells permitted their continuous propagation. The growth of irradiated SHE cells depended on the presence of rhTNF-α only within the first 30 passages, after which the cells could be subcultured in the absence of rhTNF-α. In contrast, both TNF-α-treated control cells and α-irradiated cells, without subsequent TNF-α treatment, senesced after 7–15 passages. It seemed that TNF-α played a critical role in the acquisition of expanded lifespan for the α-irradiated SHE cells.

An anti-TNF-α antibody could be used to prevent binding of TNF-α to its receptor and to promote clearance of TNF-α from the circulation via antigen–antibody complexes (Engelmann et al, 1990). In our experiment, the TF decreased significantly in the presence of a 2 μg $ml^{-1}$ dose of anti-TNF-α antibody that could neutralize the cytotoxicity due to 600 U $ml^{-1}$ TNF-α. However, the TF was still much higher than that of cells exposed to a 0.5 Gy dose of α-particles alone. This suggested the presence of other known or unknown effectors that could act as tumour promoters in this supernatant. On the other hand, it cannot be excluded that feeders may release something that may cooperate with TNF-α to increase the transformation frequency.

© Cancer Research Campaign 1998

In summary, the present study provides the first evidence that administration of the endogenous inflammatory agent TNF-α promotes malignant transformation induced by exposure to α-particles. Our data suggest that TNF-α, released by activated macrophages in and around areas of chronic inflammation, may be an important contributor to tumour promotion.

### ACKNOWLEDGEMENTS

We would like to thank Dr Tom K Hei, Center for Radiological Research, Columbia University, for his suggestions for revising this manuscript, and his encouragement, Dr L Hieber and Professor PeiFen Shen for providing hostaphan membrane and anti-TNF antibody. This study was supported by Chinese National Science Grant No. 39170273.

### REFERENCES

Bennett SAL, Leite LCC and Birndoim HC (1993) Platelet activating factor, an endogenous mediator of inflammation, induces phenotypic transformation of rat embryo cells. *Carcinogenesis* **14**: 1289–1296

Borek C and Hall EJ (1973) Transformation of mammalian cells in vitro by low doses of X-rays *Nature* **243**: 450–453

Borek C and Troll W (1983) Modifiers of free radicals inhibit in vitro the oncogenic actions of X-rays. bleomycin, and the tumor promoter 12-O-tetradecanoylphorbol-13-acetate. *Proc Natl Acad Sci USA* **80**: 1304–1307

Crawford-Brown DJ and Shyr L-J (1987) The relationship between hit probability and dose for alpha emissions under selected geometries. *Radiat Protect Dosimet* **20**: 155–168

Dipaolo JA (1980) Quantitative in vitro transformation of syrian hamster embryo cells with the use of frozen stored cells. *J Natl Cancer Ins* **64**: 1485–1494

Donaldson K, Li XY, Dogra S, Miller BG and Brown GM (1992) Asbestos-stimulated tumour necrosis factor release from alveolar macrophages depends on fiber length and opsonization. *J Pathol* **168**: 243–248

Engelmann H, Holtmann H, Brakebusch C, Avni YS, Sarov I, Nophar Y, Hada E, Leitner O and Wallach D (1990) Antibodies to a soluble form of a tumor necrosis factor (TNF) receptor have TNF-like activity. *J Biol Chem* **265**: 14497–14504

Floyd R (1990) Role of oxygen free radical in carcinogenesis and brain ischemia. *FASEB* **4**: 2587–2597

Fujiki H and Suganuma M (1994) Tumor necrosis factor-alpha, a new tumor promoter, engendered by biochemical studies of okadaic acid. *J Biochem* **115**: 1–5

Galloway CJ, Madanat MS and Mitra G (1991) Monoclonal anti-tumor necrosis factor (TNF) antibodies protect mouse and human cells from TNF cytotoxicity. *J Immunol Methods* **140**: 37–43

Hieber L, Ponsel G, Roos H, Fenn S, Fromke E and Kellerer AM (1987) Absence of a dose-rate effect in the transformation of C3H10T1/2 cells by α -particles. *Int J Radiat Biol* **52**: 859–869

Hu LP, Gong YF and Wu DC (1989) Damage of membrane of rat alveolar macrophage by irradiation in vitro. *J Radiat Res Process* **2**: 15–20

Jiang WG, Puntis MCA and Hallett MB (1992) U937 cells stimulated with opsonised zymozan particles provide a convenient laboratory source of tumor necrosis factor α. *J Immunol Methods* **152**: 201–207

Komori A, Yatsunami J, Suganuma M, Okabe S, Sakai A, Sasaki K and Fujiki H (1993) Tumour necrosis factor acts as a tumor promoter in BalB/3T3 cell transformation. *Cancer Res* **53**: 1982–1985

Lloyd EL, Gemell MA, Henning CB, Gemmell DS and Zabransky BJ (1979) Transformation of mamalian cells by alpha-particles. *Int J Radiat Biol* **36**: 467–478

Mohr C, Gemsa D, Graebner C, Hemenway DR, Leslie KD, Absher PM and Avis GS (1991) Systemic macrophage stimulation in rats with silicosis: Enhanced release of tumor necrosis factor-α from alveolar and peritoneal macrophages. *Am J Respir Cell Mol Biol* **5**: 395–402

Mosmann T (1983) Rapid colorimetric assay for cellular growth and survival: application to proliferation and cytotoxicity assays. *J Immunol Methods* **65**: 55–63

NCRP Report No. 78 (1984) *Evaluation of occupational and Environmental Exposure to Radon and Radon Daughters*. National Council on Radiation Protection and Measurement: Washington, DC

Old LJ (1985) Tumor necrosis factor (TNF). *Science* **230**: 630–632

Owens MW, Milligan SA and Grisham MB (1995) Nitric oxide-dependent N-nitrosating activity of rat pleural mesothelial cells. *Free Radic Res* **23**: 317–318

Pienta RJ, Lebherz WB and Schuman RF (1981) The use of cryopreserved syrian hamster embryo cells in a transformation test for detecting chemical carcinogens. In *Short-Term Tests for Chemical Carcinogens*, Stich HF and San RHC (eds), pp. 323–360. Springer: New York

Roberts CJ and Goodhead DT (1987) The effects of $^{238}$Pu α particles on the mouse fibroblast cell line C3H10T1/2: characterization of source and RBE for cell survival. *Int J Radiat Biol* **52**: 871–882

Suganuma M, Okabe S, Sueoka E, Iida N, Komori A, Kim SJ and Fujiki H (1996) A new process of cancer prevention mediated through inhibition of tumor necrosis factor alpha expression. *Cancer Res* **56**: 3711–3714

Suzuki F, Suzuki K and Nikaido O (1983) An improved soft agar method for determining neoplastic transformation in vitro. *J Tissue Culture Methods* **8**: 109–113

Suzuki K, Suzuki F, Watanabe M and Nikaido O (1989) Multistep nature of x-ray-induced neoplastic transformation in golden hamster embryo cells: Expression of transformed phenotypes and stepwise changes in karyotypes. *Cancer Res* **49**: 2134–2140

Templeton AC (1975) Acquired diseases. In *Persons at High Risk of Cancer: An Approach to Cancer Etiology and Control*, Fraumeni JF (ed.), pp. 69–84. Academic Press: New York

Thomassen DG, Seiler FA, Shyr LJ and Griffith WC (1990) Alpha-particles induce preneoplastic transformation of rat tracheal epithelial cells in culture. *Int J Radiat Biol* **57**: 395–405

Tomkins P, Cooper K, Webber D and Bowen G (1992) The L929 cell bioassay for murine tumour necrosis factor is not influenced by other murine cytokines. *J Immunol Methods* **151**: 313–315

Vassalli P (1992) The pathophysiology of tumor necrosis factor. *Annu Rev Immunol* **10**: 411–552

Walsh PJ (1970) Stopping power and range of alpha particles. *Health Physics* **19**: 312–316

Xie GL, Yan XS, Wu DC, Ye CQ, Guo LS, Gong YF, Guan SR and Wang RZ (1989) Dose-effect relationship of carcinogenic effect of inhaled $^{239}PuO_2$ on rat lungs. *Radiation Protection* **15**: 157–162

Zhang X, Zheng WZ, Gong YF, Li WB and Yang ZH (1996) An α irradiation model for radiobiological studies. *Radiation Protection* **16**: 192–202

Zimmerman R and Cerutti P (1984) Active oxygen acts as a promoter of transformation in mouse embryo C3H/10T1/2/C18 fibroblast. *Proc Natl Acad Sci USA* **81**: 2085–2087

© Cancer Research Campaign 1998